# Advances in the Study of Heartwood Formation in Trees

**DOI:** 10.3390/life15010093

**Published:** 2025-01-14

**Authors:** Shuqi Yang, Fangcuo Qin, Shengkun Wang, Xiang Li, Yunqing Zhou, Sen Meng

**Affiliations:** State Key Laboratory of Tree Genetics and Breeding, Research Institute of Tropical Forestry, Chinese Academy of Forestry, Guangzhou 510520, China; yshuqi@caf.ac.cn (S.Y.); qinfc@caf.ac.cn (F.Q.); wskun2001@163.com (S.W.); lx541307@163.com (X.L.); zhouyunqing824@163.com (Y.Z.)

**Keywords:** heartwood, sapwood, vascular cambium, programmed cell death, secondary metabolites

## Abstract

Heartwood, serving as the central constituent of the xylem, plays a crucial role in the growth, development, and resilience of trees. The process of heartwood formation constitutes a complex biological phenomenon influenced by various factors. A thorough examination of the mechanisms underpinning heartwood formation not only enhances our understanding of the growth and developmental paradigms regulating trees but also provides essential theoretical support and practical insights for the timber industry, forestry management, and ecological conservation. This paper offers an overview of the foundational processes involved in heartwood formation in plants. Furthermore, it presents a comprehensive review of the latest research advancements in this domain, covering five key aspects: metabolism, hormonal regulation, transcriptional regulation, cell biology, and environmental influences. This review serves as a valuable basis for future research endeavors in related academic fields.

## 1. Introduction

**Figure 1:**
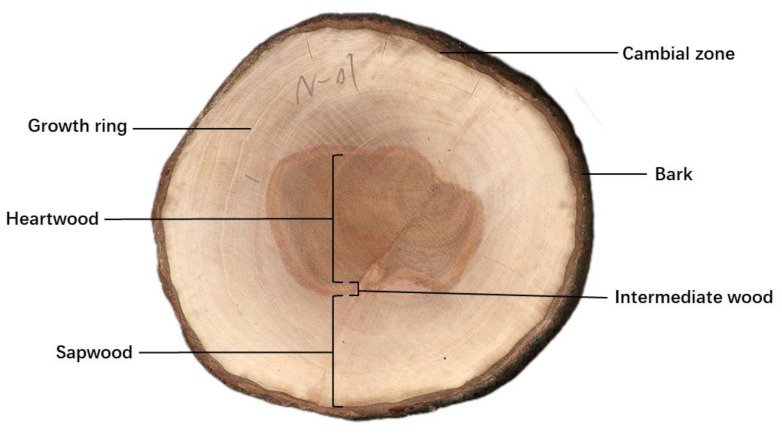
The location of heartwood, transition zone, sapwood, growth ring, cambial zone, and bark in the sandalwood disc is demonstrated, with brown heartwood in the centre and light sapwood in the periphery.

Wood is comprised predominantly of secondary xylem, a vital tissue that fulfills the dual role of providing mechanical support and facilitating water conduction [1]. The production of secondary xylem is attributed to the activity of the cambial zone, a lateral meristem situated between the primary xylem and phloem. The cambial zone is responsible for the diameter growth of tree axes, both in the shoot and the root, through its continuous cell division and subsequent differentiation. Newly formed cells are attached to the outer layer of the primary xylem. This contributes to the radial expansion [2]. As the cambial zone expands outward, secondary xylem undergoes expansion and thickening, resulting in an augmentation of stem diameter [3]. Sapwood (SW) is adjacent to the inside of the cambial zone, while the heartwood (HW) is adjacent to the inside of the sapwood. And the intermediate wood is present between the heartwood and sapwood. They can usually be identified in tree disc by their respective colors [4,5] (Figure 1). The sapwood, characterized by its lighter color and greater porosity, contrasts with the heartwood, which is darker in color and more compact in structure [1,6]. The intermediate wood acts as a transition zone (TZ) between the middle sapwood and the heartwood. In some instances, the TZ can be distinguished by its white color. This phenomenon occurs when water loss occurs in the intermediate wood region during heartwood formation [7].

Sapwood (SW) represents the secondary xylem generated by the vascular cambium during the later life stages of a tree [8]. It is characterized by a relatively low accumulation of substances such as resins, gums, and tannins [9]. Heartwood is earlier-formed secondary xylem; both angiosperms and gymnosperms have heartwood [10]. The sapwood (SW) remains active for a finite duration, subsequently transitioning into heartwood (HW). At the micro level, vessels and tracheids are present in secondary xylem, which are responsible for conducting water and nutrients, as well as wood parenchyma cells and wood rays, which serve functions in nutrient storage and transport [11]. Gymnosperms characteristically lack vessels, except in Gnetopsidas, where some species exhibit vessel presence [12]. During the process of heartwood formation, there is an explosive increase in cellular metabolic activity in the transition zone [13,14], during which primary metabolism proceeds rapidly, resulting in substantial nutrient accumulation. This process leads to the accumulation of secondary metabolites by the parenchyma cells in the sapwood, which subsequently undergo programmed cell death (PCD) to form HW tissue [15,16,17,18]. Vessels and tracheids (or tracheids only) within HW lost their conductive capabilities due to the formation of tyloses from the dead parenchyma cells, which block the vessel pores as a result of metabolic processes [19]. Consequently, HW forms a hard cylinder composed of dead cells, with an accumulation of various secondary metabolites (woody known as extractives) [13]. These extractives usually exhibit distinctive coloration, resulting in a darker hue for HW that can be readily discerned from SW.

Nevertheless, there are certain tree species whose sapwood exhibits minimal differentiation from the heartwood, rendering color shade classification ineffective. This phenomenon typically appears in gymnosperm trees, including *Schima superba*, *Picea asperata*, and *Abies fabri*. These trees are also referred to as “sapwood trees”. The formation of these trees is attributed to the presence of extractives that are white or colorless in color [20]. The occurrence of wood discoloration has been documented in various angiosperm species, such as poplar, beech (*Fagus sylvatica*), wild cherry (*Prunus avium*), paper birch (*Betula papyrifera*), silver birch (*Betula pendula*), and ash (*Fraxinus excelsior*). This discoloration phenomenon has been observed in some species that are traditionally considered to lack heartwood [21]. This type of weakly differentiated heartwood, also referred to as false heartwood, exhibits minimal pigmentation and is not durable [22].

As discussed previously, relying solely on color to delineate the range of heartwood and sapwood is insufficient for achieving rigor in description. A more rigorous approach entails a microscopic observation of the parenchyma cells in different parts, including the number of starch grains and the different shapes of their nucleus [23]. Then, based on the distribution of parenchyma cells in different states, different zones are determined. The width of heartwood is influenced by the width of the growth rings (annual rings), which tends to be narrower in older trees. It is more pragmatic to utilize the number of growth rings to characterize the extent of heartwood and sapwood when representing the physiological activity of heartwood-forming trees [7].

The qualities and characteristics of heartwood have a direct impact on the utilization and economic worth of woods, as well as the extent to which trees adapt to their environment. Wood with a higher proportion of heartwood is harder, denser, and more suitable for furniture and construction materials. Due to the presence of numerous secondary metabolites in heartwood, it has strong resistance to decay, insect damage, and pathogens [17]. However, the high lignin content and hardness of the tissue pose additional obstacles to experimental manipulation of the plant material. The composition of heartwood (HW) includes a substantial number of dead cells, which significantly compromises the quality of RNA extracted from HW, rendering the extraction process difficult. Furthermore, heartwood formation is a gradual process, making it impractical to observe an entire life cycle within a short timeframe. Collectively, these factors render the investigation of the molecular mechanisms underlying heartwood formation a formidable task. The majority of current research on the mechanisms of heartwood formation in trees has been confined to the omics level (transcriptomics, proteomics, metabolomics), and further in-depth studies are necessary to elucidate the functional roles of specific regulation pathways. This represents a crucial direction for future research endeavors in this field.

## 2. Mechanisms and Regulation of Heartwood Formation

The development of heartwood constitutes a complex physiological process influenced by a wide array of internal and external factors. The investigation of heartwood formation mechanisms can be approached from various perspectives, including metabolism, hormonal regulation, transcriptional control, cellular biology, and environmental impacts.

### 2.1. Metabolism

Increased metabolic activity of parenchyma cells during heartwood formation, with primary metabolism providing precursors and energy for metabolic activity [24]. A comprehensive analysis of the *D. odorifera* genome has revealed a significant up-regulation of numerous genes implicated in fundamental metabolic processes within the TZ, including those encoding sucrose synthase (SS) and sucrase (INV), which catalyze the conversion of sucrose, along with the genes of the key regulatory enzyme, 3-deoxy-D-arabinopyranosiduronic acid-7-phosphate synthase (DHAPS), within the mangiferolic acid pathway [25]. Heartwood extractives are secondary metabolites, predominantly phenolic compounds, which are responsible for the pigmentation, biological (natural durability), technological, and commercial properties of heartwood and its products [26]. Heartwood extractives show a large variety of different chemical structures and thus belong to different chemical classes such as tannins, terpenes, flavonoids, stilbenes, lignins, aromatic substances, and lectins [15]. Lignin is one of the main components that determines the quality of wood, and the process of lignification of the cell wall during heartwood formation is accelerated in the transition zone [9,27]. Lignin is a critical factor in determining woods’ natural durability. This polymer plays a pivotal role in plant biology, contributing to the rigidity of cell walls and enhancing water repellency. It also functions as a protective barrier against microbial degradation [28]. However, the chemical reactivity of heartwood extractives with pre-existing cell wall components is poorly understood. It seems likely that cell wall infiltration involves a mechanism of enzymatically initiated but chemically driven copolymerization between these phenolic derivatives and the pre-existing cell wall macromolecular components, including lignins and lignin-polysaccharide complexes [15].

The formation of sandalwood heartwood is accompanied by the accumulation of sandalwood oil, a complex mixture of terpenes and terpenoids [29,30]. Sandalwood essential oil is predominantly constituted of the sesquiterpene alcohols α-, β-, and epi-β-santalol, as well as α-exo-bergamotol, accounting for approximately 90% of its chemical composition [31]. The formation of these disparate components is attributable to the activity of SaSSY (a terpen synthase enzyme) and the oxidation of sandalwood cytochrome P450 oxygenase [29]. Ma et al. analyzed the composition of *Dalbergia odorifera* heartwood using UPLC-MS. The flavonoids, which are characteristic of the heartwood, are key compositions in the color formation of the wood. The characteristic flavonoid compositions were highest in the xylem of the heartwood, with the transition zone being the place where the heartwood compositions begin to accumulate in large quantities [23]. The scent and color of *D. odorifera* are primarily attributable to its sesquiterpenes and flavonoids. In addition, its flavonoids demonstrate a wide range of biological and pharmacological activities [32]. *D. odorifera* has historically been utilized in traditional Chinese medicine for the treatment of cardiovascular diseases, blood disorders, and ischemia, as well as pain management [33]. Dag et al. utilized gas chromatography (GC) with flame ionization detection (FID) to quantitatively determine extractives from the heartwood of Scots pine (*Pinus sylvestris* L.). The analysis revealed the presence of pinosylvin, pinosylvin monomethyl ether, resin acids, and free fatty acids, which were extracted from P. sylvestris heartwood [34]. It has been demonstrated that certain extractives enhance the natural wood’s resilience against deterioration by microorganisms and insects. Pinosylvin, a phenolic compound and stilbene, has demonstrated a substantial impact on the durability of natural wood and has been shown to exert a restraining effect on fungi [35].

### 2.2. Hormonal Influences

Current research on the role of hormones in the formation of tree heartwood is relatively scarce. Ethylene has been established as a crucial regulator of heartwood formation. Studies have demonstrated that the application of ethylene significantly enhances the area of heartwood in branches of *D*. *odorifera* while concurrently stimulating the biosynthesis of secondary metabolites within the heartwood [15]. Similarly, the effectiveness of ethylene treatment in promoting heartwood formation has been documented [27,28]. Additionally, Li et al. reported that 6-benzyladenine (BA), a chemoattractant, effectively induced heartwood formation in 5-year-old sandalwood trees. This finding suggests that cytokinin, specifically BA, also plays a favorable role in the development of sandalwood heartwood [29]. In Acacia melanoxylon, the process of HW formation occurs within the TZ of the subject, and its relationship to phytohormones is a subject of current research. The levels of gibberellin (GA), jasmonic acid (JA), salicylic acid (SA), and citokinin (CTK) in the TZ sample of A. melanoxylon were all found to be significantly higher than in the SW and HW samples. Furthermore, the levels of ethene (ETH) were found to be notably higher in the TZ and HW samples, indicating their potential role in heartwood synthesis [36].

The plant hormones ethylene and auxin have been postulated to be intimately associated with HW formation. According to the research by Beauchamp on *Sitka spruce* and *Scots pine*, auxin levels were either reduced or below the level of detection in the TZ. These findings indicate that depletion of auxin content is associated with HW formation [37]. The plant hormones ethylene and auxin have been suggested to be involved in HW formation, with their ratio being particularly important [26]. However, Kean-Jin’s research on Scots pine did not support this hypothesis [10]. The enzyme category of chitinases has been demonstrated to be implicated in developmental processes, including the process of secondary cell wall lignification. This is a pivotal process in the formation of heartwood, a key feature of the tree’s anatomy. The induction of these enzymes is stimulated by plant hormones [38].

### 2.3. Transcriptional Regulation

Transcription factors (TFs) are indispensable regulatory proteins in plants, executing their gene regulatory functions through interactions with localized or distant cis-elements of genes, commonly known as DNA binding sites [39]. Lim et al. [14] observed a significant upregulation of Myeloblastosis Proto-Oncogene Transcription Factor (MYB) and NAM ATAF1/2 CUC2 Transcription Factor (NAC) in the transition zone (TZ) compared to the sapwood (SW), with expression levels being 12-fold and 6-fold higher, respectively. The MYB TF family has been recognized as a pivotal regulator of secondary metabolite pathways [40]. Ma et al. [41] conducted a genome-wide analysis of R2R3-MYB TFs in *D. odorifera* and identified DodMYB89 as a potential regulator of secondary metabolite biosynthesis in heartwood, as evidenced by its ability to activate the promoters of structural genes *DodI2’H* and *DodCOMT*. Additionally, NAC-structural domain proteins have been implicated in secondary cell wall biosynthesis and programmed cell death (PCD), processes that are also linked to heartwood formation [10]. Zhang et al. [36] analyzed metabolic and RNA-seq data from Acacia melanoxylon and identified key TFs regulating the accumulation of flavonoids and phenolics. They are the heartwood extractives of *D. odorifera*, serving as the source of color and medicinal value of *D. odorifera* heartwood and protecting the wood itself from corrosion and disease. Their findings revealed TFs with contrasting correlations, such as WRKYGQR DNA Binding Domain with Zinc Finger Structure Transcription Factor (WRKY) and MYB, as well as those with positive correlations, including APETALA2/Ethylene Responsive Transcription Factor (AP2), Basic leucine Zipper Transcription Factor (bZIP), C-repeat binding Transcription Factor (CBF), Phox and Bem1 Transcription Factor (PB1), and Teosinte branched1 *Cycloidea Proliferation* Cell Factor 1/2 Transcription Factor (TCP). In Meng’s research on *Santalum album* [29], a marked increase in sandalwood ABA-responsive Element Binding protein (AREB) TF expression under drought conditions was observed, paralleled by elevated levels of santalols. Given the correlation between heartwood formation and secondary metabolites, it is plausible to hypothesize that TFs involved in secondary metabolite biosynthesis play a crucial role in the process of plant heartwood formation.

### 2.4. Cell Biology

Morphological alterations in cells have been documented during the process of heartwood formation. During heartwood formation, parenchyma cells undergo an increase in metabolic activity, which is supported primarily by primary metabolism, thereby providing precursors and energy for this metabolic activity [24].Lim et al. [10] conducted a transcriptome analysis of *Scots pine* and identified several significant findings. Firstly, they observed an upregulation of transcripts encoding two enzymes related to the program cell death (PCD), S1-like nuclease and bifunctional nuclease (BFN), in the transition zone by 13-fold and 20-fold, respectively, compared to the SW. This indicated that PCD occurred, initiated from TZ. Subsequently, transcripts encoding enzymes involved in lignification were found to be upregulated by an average of 4- to 16-fold in the TZ. Conversely, transcripts associated with cell wall carbohydrate modification exhibited an average increase in expression of 12-fold in the TZ compared to the SW. Means that lignification of the cell wall has occurred in TZ. Lignin deposition occurs when plant cells complete secondary wall expansion and form an interwoven network with cellulose and hemifibres to harden the cell wall [42]. However, it is important to note that parenchymal cells can survive and function for several years despite the presence of a lignified secondary cell wall, suggesting that programmed cell death is not directly related to lignification [22].

In the majority of gymnosperms, the living cells of the SW progressively undergo PCD, extending into the TZ. In contrast, in most angiosperms, ray parenchyma cells remain viable until the innermost SW tissue, experiencing a sudden and widespread PCD in the TZ region [15]. When PCD occurs, the vesicular membrane dissociates and releases specific hydrolases (Cys and Ser proteases, nucleases, and RNase). In addition, the pH of the cell membrane drops dramatically, activating cell membrane hydrolases. The nucleus, plasmids, and mitochondria disappear, and the cytoplasm is emptied [22]. As PCD progresses, the nutrients stored in the parenchyma cells are depleted [17,43]. The abundance of starch grains in the outer SW decreases gradually towards the TZ, with a significant reduction observed in the middle SW [44]. Phosphatases catalyze the hydrolysis of starch in the middle SW, resulting in the formation of sugar phosphates [45]. These phosphates then serve as precursors for the synthesis of lipids or secondary metabolites. The depletion of nutrients and the accumulation of secondary metabolites further contribute to cellular aging and death [46,47]. In angiosperm trees, dead parenchyma cells close to vessels are transformed into balloon-like structures called tyloses, which develop when turgor pressure causes part of their protoplast to expand outward through a pair of pits into the lumen of an adjacent vessel cell [5]. The factor that results in a decrease in the water content of sapwood is the obstruction of vessels by tyloses [15]. In gymnosperms, tyloses are almost absent, only in the resin canals of a few genera, especially Pinus, or in the tracheids of a few genera, but only as a result of wounding [48]. However, the loss of moisture remains the remarkable characteristic for coniferous heartwood formation. Katsushi et al. utilized Cryo-scanning electron microscopy to examine the water distribution within the xylem of Cryptomeria japonica. Their findings revealed that tracheids within the intermediate wood are devoid of water. This suggests that loss of water is important in the process of SW-HW transformation [49].

Song et al. [46] performed an anatomical analysis of the stem of *Cunninghamia lanceolata*, enabling the observation of the different states of tracheids (T) and ray parenchyma (R) cells in them. Changes of these two cell types in the formation of heartwood of *C. lanceolata* were then summarized. They observed a marked thickening of the cell walls of tracheids as they pro-gressed from the intermediate wood to the middle heartwood. From the intermediate sapwood to the heartwood, the protoplasmic bodies of the xylem tracheids underwent complete degradation, transforming into hollow dead cells. The protoplasts of xylem ray cells were observed to be smaller and more dispersed compared to those of cambium ray cells. Furthermore, the number of organelles (such as mitochondria, endoplasmic reticulum, and Golgi apparatus) decreased gradually from the cambium zone to the intermediate wood. Simultaneously, the shape of the nucleus underwent changes, transitioning from spherical to oval and then to spindle-shaped. The cellular starch content decreased from the cambium zone towards the intermediate wood, while the number of lipid droplets increased initially from the cambium zone to the middle sapwood and then decreased towards the intermediate wood. All ray parenchyma cells in the heartwood had lost their protoplasm. In *Dalbergia odorifera*, Ma et al. [23] observed the presence of parenchyma cells in the xylem. They found that, as the region moved from SW to TZ, the content of starch granules in parenchyma cells decreased rapidly, and in the HW region, starch granules almost disappeared. From SW to HW, the shape of nuclei in parenchyma cells gradually shortened and rounded from oval shape and finally disappeared. Interestingly, this is diametrically opposed to Song’s observations in C. lanceolata, suggesting differences in apoptotic pathways of parenchyma cells between gymnosperms and angiosperms. One study has posited the hypothesis of the existence of living parenchyma cells in the heartwood tissues of sandalwood. These cells are specialized in the synthesis of secondary metabolites, continuing metabolic activities in the heartwood zone, while other cells undergo normal programmed cell death [17]. However, Satish et al. did not observe oil-containing cells between axial or radial parenchyma and fibers during their anatomical examination of sandalwood heartwood tissues. Instead, they noted the presence of tiny brownish-yellow oily particles in the lumens between procumbent ray cells and axial parenchyma. Additionally, vessels were found to contain oil globules, as previously reported by Susikumar [50].

### 2.5. Environmental Influences

The process of heartwood formation is intricate and varies between species. This is evidenced by the substantial variation in heartwood characteristics observed among different species. Such variation can be attributed to the diverse genetic backgrounds inherent within these species. In the case of Pinus canariensis, the variation in heartwood was predominantly attributed to age and initial growth patterns, with 51–73% of the observed variations being explained by these factors. The onset of heartwood formation is not a singular event that occurs at a specific age but rather, it emerges when the tree reaches a certain diameter [51]. A total of 30% of trees had red heartwood at the age of 80 years in contrast to 95% over 180 years old [52].The environment in which a tree grows exerts a significant influence on its growth pattern, which, in turn, has a profound effect on the progression of heartwood formation. Researchers have determined that larger trees, trees with slower growth rates, and trees growing in more acidic areas tend to have larger heartwood areas. In contrast, trees growing in more alkaline soil (i.e., pH ranging from 4.8 to 6.0) tend to have smaller heartwood [53,54,55]. Consequently, industry practitioners (e.g., timber buyers, mills, loggers) can often predict the heartwood content of these trees based on information about their growth background [56]. In Peter et al.’s investigation of Sugar Maple heartwood-soil relationships [56], they observed that larger areas of red heartwood were found primarily in soils with incomplete or poor drainage, limited lateral water movement, and acidic (granite-dominated) parent material. Ryogo et al. [57] revealed that the formation of Larix kaempferi heartwood is subject to seasonal variation. Firstly, during the transition from sapwood to intermediate wood, the lumen of tracheids undergoes a loss of water; secondly, from April to July, when the cambium is most active, ray parenchyma cells undergo death; and finally, the newly produced heartwood is deposited in late autumn and early winter, i.e., the transition from mesocarp to heartwood. Environmental moisture has been recognized as a factor influencing heartwood formation. Julian et al. compared heartwood development in radiata pine trees from a high-altitude subalpine region to those from a warm, dry inland region. They observed that heartwood development was faster and extensive cavitation was more prevalent at the sapwood/heartwood interface in trees from the warmer and drier regions compared to those from higher altitudes [38]. The formation of heartwood can be conceptualized as a physiological response to changes in moisture levels within the tree. This process is a regulatory mechanism that maintains a constant and optimal proportion of functional sapwood in the trunk [58,59]. The conversion of sapwood to heartwood is a process that is regulated by the tree itself. In species subjected to prolonged and severe droughts, this ratio is an important criterion for survival [59,60]. Cui et al. further found that drought could promote heartwood formation in *D. odorifera* T. Chen by altering the proportions of non-structural carbohydrate (NSC) components [39].

It was mentioned above that ethylene gas has been demonstrated to be an effective agent in promoting the formation of heartwood. Meanwhile, Taylor et al. [61] suggested that high concentrations of CO_2_ and low concentrations of O_2_ promote the transition from sapwood to heartwood, but the observations were incomplete. Based on oxygen concentrations of less than 1% measured in the sapwood of *Picea abies* [62], it has been suggested that the hypoxic environment within the sapwood is responsible for parenchyma cell death [63]. However, Spicer and Holbrook [64] found that the oxygen content of the sapwood decreased from the outer sapwood to the inner sapwood, but the concentration was not high enough to damage the parenchyma cells. Then, Johannes and Peter [52] analyzed the sapwood of *Fagus sylvatica* and concluded that although oxygen is involved in the formation of red heartwood, it is not the only triggering factor and that microbial activity may be another important factor. Some studies have reported the presence of bacteria and fungi in the heartwood of certain tree species, and a color change similar to heartwood has been observed in the sapwood of some trees infected by fungi [40,41]. Based on these observations, a hypothesis has been proposed suggesting that fungal infection may alter the internal metabolic environment of sapwood, leading to the deposition of heartwood material and the formation of heartwood.

Increment borings and analogous lesions in the boles of forest trees give rise to vertical discoloration within the sapwood. Such discolored regions are commonly designated as wound or pathological heartwood [65]. Wound heartwood can be developed following injury to the cambium/phloem tissues. As the color of wound heartwood is very similar to that of naturally occurring heartwood, some studies have suggested that this is an extension of the normal part of the heartwood into the sapwood. Nevertheless, there remain distinctions between wound heartwood and normal heartwood. The former is incapable of being enlarged regularly and centrifugally in the manner of normal heartwood, and the latter possesses a lower pH and higher moisture and mineral content [66]. In their study, Anni et al. [67] utilized 3-year-old *Pinus sylvestris* seedlings that had been wounded by drilling holes through the stem to investigate the effects of damage on plant stems. The results demonstrated an increase in the concentration of resin acids (RAC) in the xylem adjacent to the wound, and the production of extractives characteristic of heartwood (stilbenes) and knotwood (stilbenes and lignans) of mature trees was induced. The induced stilbenes were identified as pinosylvin (PS) and pinosylvin mono-methyl ether (PSM), while the lignans detected were nortrachelogenin (NTG) and matairesinol (MR). Thus, it appears that although wound heartwood is not identical to normal heartwood, damage can promote the production of heartwood extractives at the wound sites. Bamber [68] hypothesizes the presence of a heartwood-inducing substance (HIS). He thought that the formation of heartwood was initiated by the transfer of HIS from the outer region of the sapwood, in proximity to the site of damage. HIS demonstrated the capacity to migrate longitudinally from the sapwood adjacent to the injury to the region above the injury, thereby producing traumatized heartwood specifically in that region. He also observed that in the region above the injury, after a period of time, the effect of HIS disappeared and normal sapwood appeared. It now appears that ‘HIS’ is more likely to be the result of a series of metabolic modulations rather than a single substance. While the pattern of change at the site of injury remains a subject of interest.

The activation of tree defense mechanisms is initiated by either a fungal infection or mechanical damage. These mechanisms are of significant importance to trees, as they enable the trees to adapt to changes in the external environment. Some long-lived species may have a defense mechanism on the sapwood that prevents decay, according to Adam et al. [61] Microbial invasion also elicits a response from such defense mechanisms. Sapwood contains a variety of compounds that have the capacity to inhibit microbial infections. The inhibitory compounds present in trees are phenolic in nature and are frequently also present in the heartwood of the respective species. Norlignans, lignans, stilbenes, and flavanones are the representative phenolic compounds. These compounds occur in negligible amounts in sound sapwood and are produced during the process of heartwood formation [69]. Therefore, the formation of heartwood in trees is also related to the activation of plant defense mechanisms [7]. The activation of defense mechanisms has been shown to result in the up-regulation of the expression of genes that control the activity of specific enzymes related to heartwood formation: glucose-6-phosphate dehydrogenase (G6PDH) and 6-phosphogluconate dehydrogenase (6PGDH). They are enzymes of the oxidative pentose phosphate pathway (OPP), which is involved in the interconversion and rearrangements of sugar phosphates [70].

## 3. Summary and Prospects

The anatomical aspects of the fundamental model of heartwood formation in trees have been delineated with greater precision. However, most studies on the metabolic pathways involved in this process remain at the histological level, and the underlying molecular mechanisms have yet to be fully characterized. The addition of ethylene has been demonstrated to effectively promote heartwood formation, whereas the impact of other hormones like gibberellin, auxin, cytokinin, Salicylic Acid, and Jasmonic Acid which have been proved to affect the formation of wood [36], on this process has been infrequently reported. The specific molecular pathways involved in hormone regulation during heartwood formation remain unclear and warrant further investigation. Research on the mechanisms underlying heartwood formation is transitioning from the traditional domains of histology and physiology to the more advanced fields of genetics and molecular biology. The research endeavors concerning the mechanisms of heartwood formation are transitioning from the traditional realms of histology and physiology towards the cutting-edge fields of genetics and molecular biology. In the future, the application of advanced omics technologies, such as spatial transcriptome profiling and single-cell sequencing, will provide a promising lens through which to gain a deeper understanding of the regulatory mechanisms underlying heartwood formation.

## Data Availability

No new data were created or analyzed in this study. Data sharing is not applicable to this article.
